# Supplementary figures and images for: Maternal TGF-β ligand Panda breaks the radial symmetry of the sea urchin embryo by antagonizing the Nodal type II receptor ACVRII
Source: PLoS Biol. 2024 Jun 24;22(6):e3002701. doi: 10.1371/journal.pbio.3002701 (PMC11239237; doi:10.1371/journal.pbio.3002701)

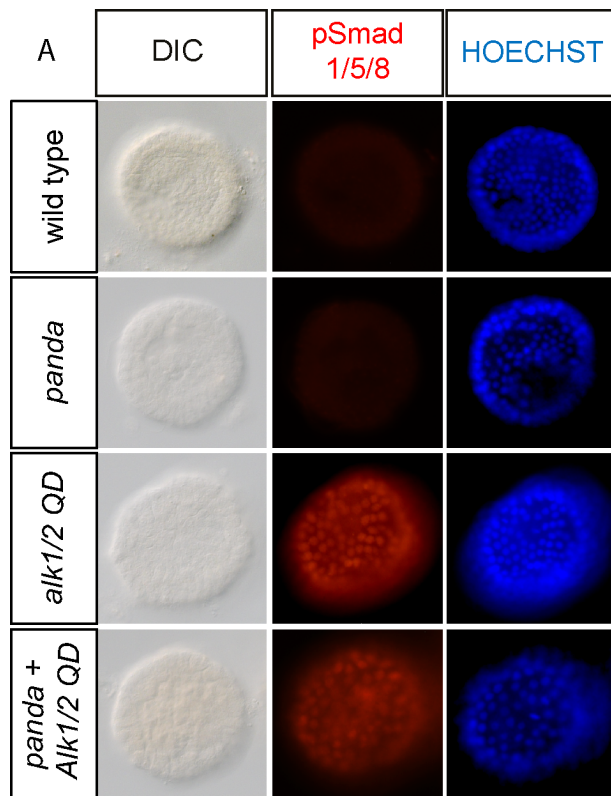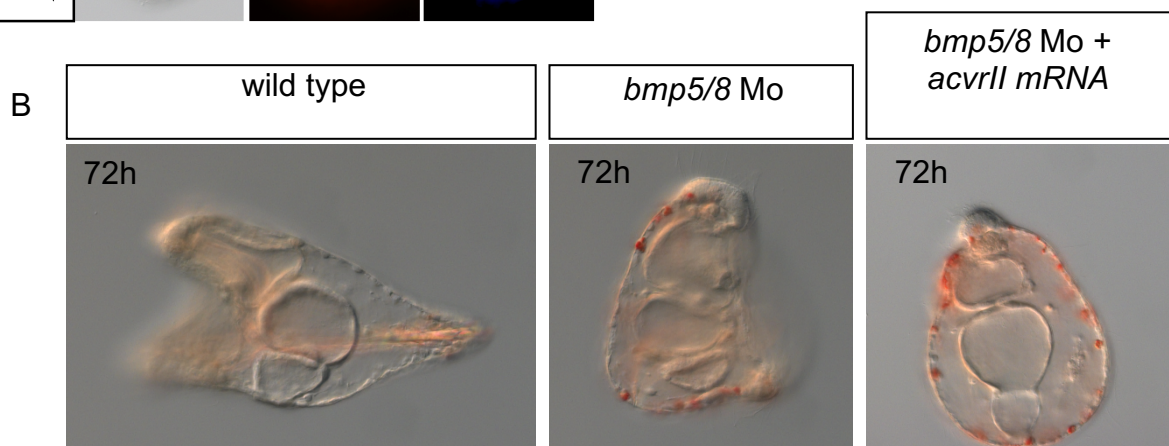

Dorsalized

**Figure S1.**

Supplement: S1 Fig — (A) Overexpression of Panda does not suppress Smad1/5/8 signaling activated by overexpression of an activated BMP type I receptor Alk1/2 QD. While overexpression of Panda suppresses BMP signaling activated by overexpression of acvrII, it does not suppress BMP signaling induced by misexpression of an activated BMP receptor. (B) Overexpression of acvrII dorsalizes embryos in the absence of BMP5/8. Figure shows wild-type embryo, embryo injected with a morpholino targeting the bmp5/8 transcript, or embryo co-injected with the bmp5/8 Morpholino and acvrII mRNA at the pluteus stage. Unlike bmp5/8 morphants, which show a typical BMP loss-of-function phenotype with an ectopic ciliary band forming on the dorsal side, embryos injected with bmp5/8 Mo and acvrII mRNA are dorsalized as indicated by their radialization, the presence of a thin ectoderm and the overpigmentation. This suggests that overexpression of acvrII dorsalizes embryos due to ligand independent activation of pSmad1/5/8 signaling. (PDF) [file pbio.3002701.s001.pdf]

Dorsal, ventral, or lateral position  
of injection clones?

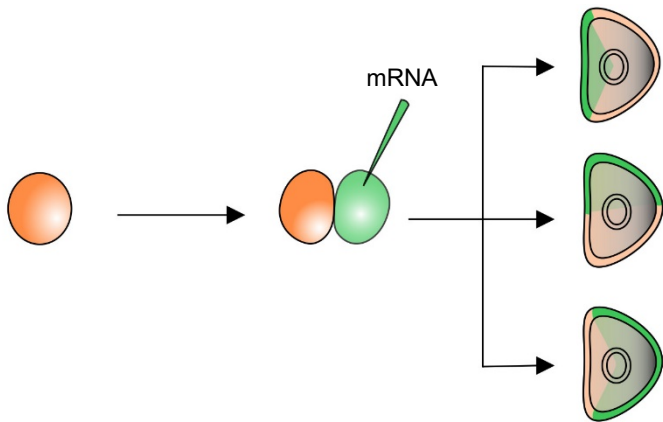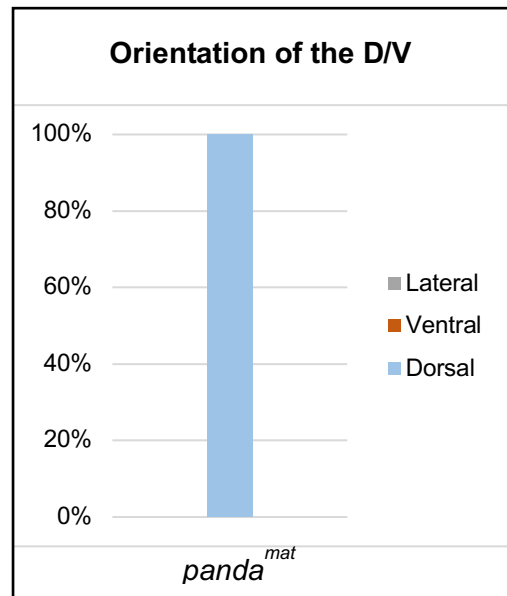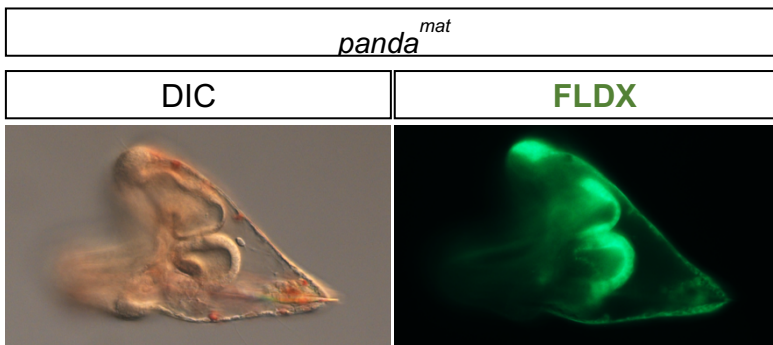

Supplement: S2 Fig — When mRNA encoding the mature form of Panda, pandamat, is injected locally into one blastomere at the two-cell stage, the progeny of the injected blastomere are on the dorsal side in 100% of the embryos injected indicating that removal of the pro-domain of Panda does not affect its activity. (PDF) [file pbio.3002701.s002.pdf]

|                         |   |   |
|-------------------------|---|---|
| Alk3/6 Myc              | + | + |
| Panda <sup>mat</sup> HA | - | + |

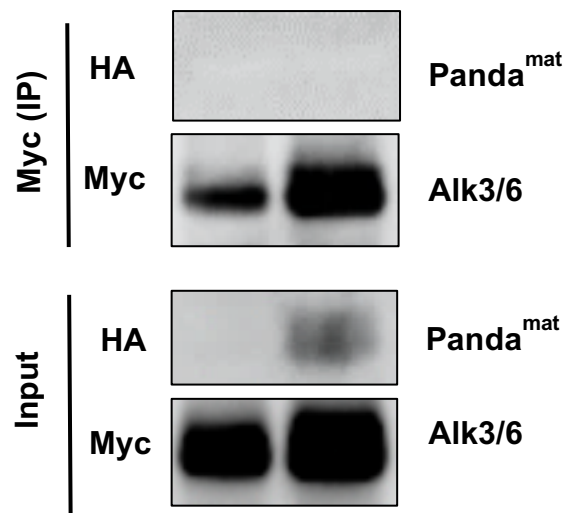

Supplement: S3 Fig — Co-immunoprecipitation of Pandamat with Alk3/6. Alk3/6 Myc co-immunoprecipitation in the presence of Pandamat when probed with anti-HA fails to detect the presence of Pandamat, showing that Panda does not interact with Alk3/6. (PDF) [file pbio.3002701.s003.pdf]

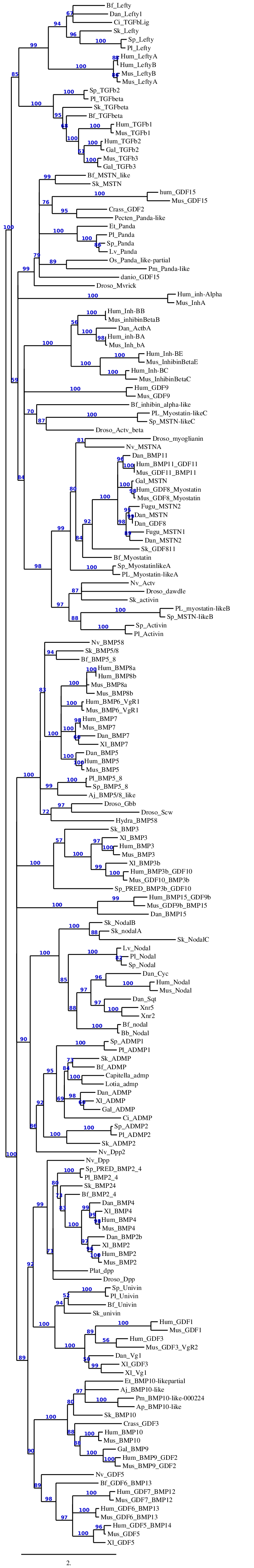

Supplement: S1 Phylogenetic Tree — Phylogenetic analysis of sea urchin and various metazoan TGF-beta ligands. The analysis was performed using the full-length proteins (see supplementary text). Maximum likelihood tree including 182 sequences. Reliability of for internal branches was assessed using the approximate likelihood ratio test (aLRT) test (SH-Like). Graphical representation and edition of the tree were performed with TreeDyn. The tree was calculated with the maximum likelihood method with PhyML with the substitution model WAG. A consensus tree with a 50% cut-off was derived using the aLRT test. (PNG) [file pbio.3002701.s009.png]
