# Supplementary material for: Maternal TGF-β ligand Panda breaks the radial symmetry of the sea urchin embryo by antagonizing the Nodal type II receptor ACVRII
Source: PLoS Biol. 2024 Jun 24;22(6):e3002701. doi: 10.1371/journal.pbio.3002701 (PMC11239237; doi:10.1371/journal.pbio.3002701)
Supplement: S4 Fig — Notice the presence of a Proline residue in the β6 region of both Panda and Lefty family members (red box) instead of a highly conserved Serine or threonine in Nodals, Activins, or BMPs (Blue box). Also notice that the region between the beta6 and beta7 strands is longer in Panda and Lefty family members compared to Nodal, Activin, and BMP ligands. (PDF) [file pbio.3002701.s004.pdf]

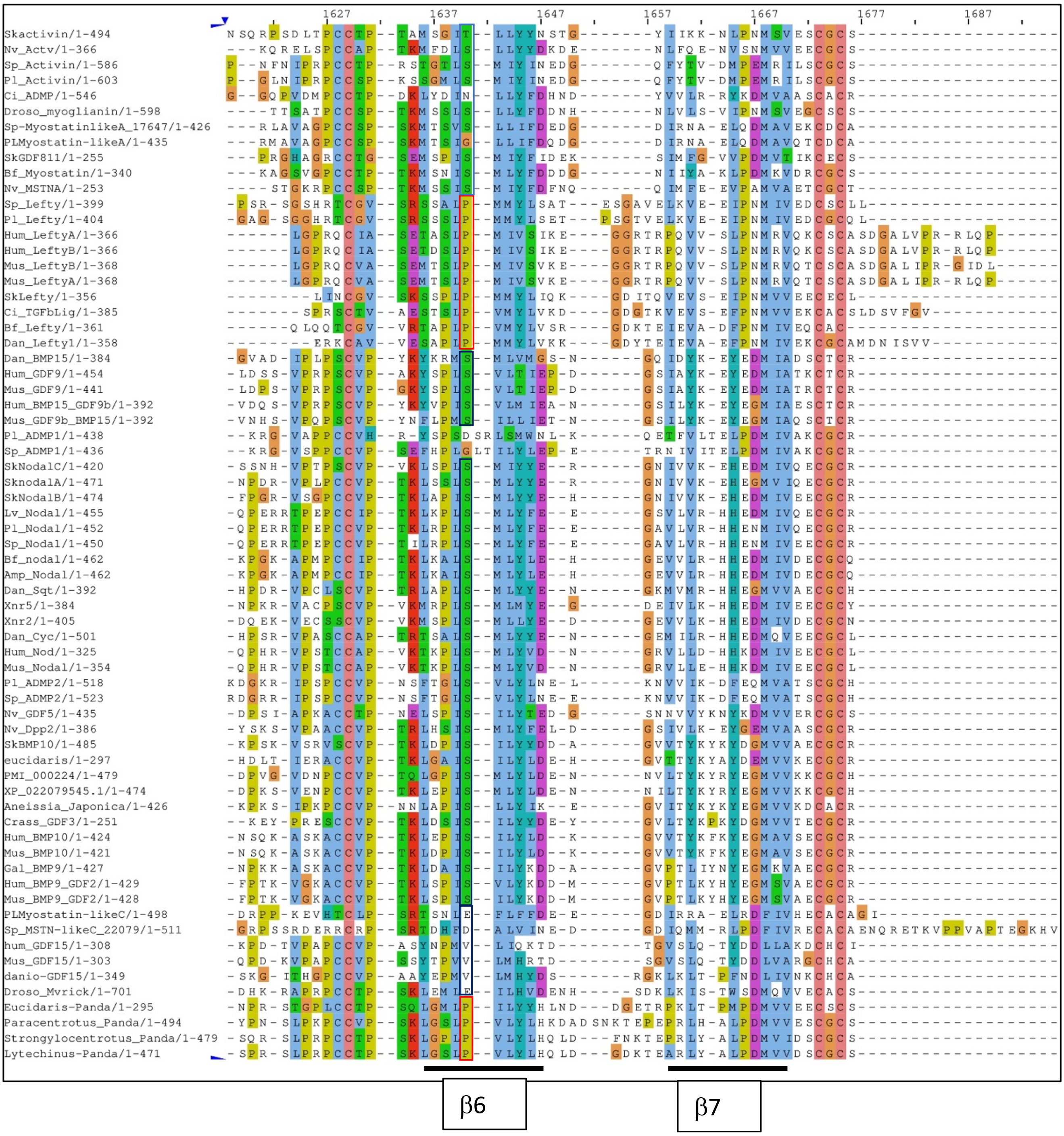

1627
1637
1647
1657
1667
1677
1687
Skactivin/1-494
Nv_Actv/1-366
Sp_Activin/1-586
Pl_Activin/1-603
Ci_ADMP/1-546
Droso_myoglianin/1-598
Sp-MyostatinlikeA_17647/1-426
PLMyostatin-likeA/1-435
SkGDF811/1-255
Bf_Myostatin/1-340
Nv_MSTNA/1-253
Sp_Lefty/1-399
Pl_Lefty/1-404
Hum_LeftyA/1-366
Hum_LeftyB/1-366
Mus_LeftyB/1-368
Mus_LeftyA/1-368
SkLefty/1-356
Ci_TGFbLig/1-385
Bf_Lefty/1-361
Dan_Lefty1/1-358
Dan_BMP15/1-384
Hum_GDF9/1-454
Mus_GDF9/1-441
Hum_BMP15_GDF9b/1-392
Mus_GDF9b_BMP15/1-392
Pl_ADMP1/1-438
Sp_ADMP1/1-436
SkNodalC/1-420
SknodalA/1-471
SkNodalB/1-474
Lv_Nodal/1-455
Pl_Nodal/1-452
Sp_Nodal/1-450
Bf_nodal/1-462
Amp_Nodal/1-462
Dan_Sqt/1-392
Xnr5/1-384
Xnr2/1-405
Dan_Cyc/1-501
Hum_Nod/1-325
Mus_Nodal/1-354
Pl_ADMP2/1-518
Sp_ADMP2/1-523
Nv_GDF5/1-435
Nv_Dpp2/1-386
SkBMP10/1-485
eucidaris/1-297
PMI_000224/1-479
XP_022079545.1/1-474
Aneissia_Japonica/1-426
Crass_GDF3/1-251
Hum_BMP10/1-424
Mus_BMP10/1-421
Gal_BMP9/1-427
Hum_BMP9_GDF2/1-429
Mus_BMP9_GDF2/1-428
PLMyostatin-likeC/1-498
Sp_MSTN-likeC_22079/1-511
hum_GDF15/1-308
Mus_GDF15/1-303
danio-GDF15/1-349
Droso_Mvrick/1-701
Eucidaris-Panda/1-295
Paracentrotus_Panda/1-494
Strongylocentrotus_Panda/1-479
Lytechinus-Panda/1-471
β6
β7
