# Supplementary material for: Maternal TGF-β ligand Panda breaks the radial symmetry of the sea urchin embryo by antagonizing the Nodal type II receptor ACVRII
Source: PLoS Biol. 2024 Jun 24;22(6):e3002701. doi: 10.1371/journal.pbio.3002701 (PMC11239237; doi:10.1371/journal.pbio.3002701)
Supplement: S1 Text — (DOCX) [file pbio.3002701.s008.docx]

**Supplementary text: Abbreviations of the names used and accession numbers of TGF-β used for the phylogeny**

**Abbreviations of the names used are the following:**

Hum: Human

Mus: *Mus musculus*

Dan: *Danio rerio*

Xl: *Xenopus laevis*

Gal: *Gallus gallus*

Ci: *Ciona intestinalis*

Bf: *Branchiostoma floridae*

Sk: *Saccoglossus kowaleskii*

Sp: *Strongylocentrotus purpuratus*

Pl: *Paracentrotus lividus*

Lv: *Lytechinus variegatus*

Et: *Eucidaris tribuloides*

Pm: *Patiria miniata*

Os: *Ophiotrix spiculata*

Aj: *Anessia japonica*

Ap: *Acanthaster planci*

Bb: *Branchiostoma belcheri*

Nv *Nematostella vectensis*

Pecten: *Pecten maximus*

Crass: *Crassostrea gigas*

Lotia: *Lotia gigantea*

Droso: *Drosophila melanogaster*

**Accession numbers of the sequences used for the phylogenetic analysis are the following:**

Vertebrata

***Homo sapiens (*human*)***

Hum_BMP2A (P12643), Hum_BMP4 (P12644), Hum_BMP10 (NP_055297), Hum_BMP11 (Q95390),Hum_BMP7 (P18075), Hum_BMP5 (P22003), Hum_BMP15 (NP_005439), Hum_BMP9 (Q9UK05), Hum_BMP6 (P22004), Hum_BMP3b (P55107), Hum_BMP3 (P12645), Hum_BMP8a (NP_861525), Hum_BMP8b (AAI08679), Hum_Nodal (AAH33585), Hum_Lefty A (O00292), Hum_Lefty B (O75610), Hum_TGFβ2 (P61812), Hum_GDF8 (O14793), Hum_GDF3 (Q9NR23), Hum_BMP10 (O95393), Hum_GDF5/BMP14 (P43026), Hum_Inhibin β C (NP_005529.1), Hum_GDF1 (AAB94786.1), Hum_GDF6/BMP13 (XP_373260), Hum_GDF7/BMP12 (NP_878248.2), Hum_GDF9 (NP_005251.1), Hum_GDF15 (NP_004855), Hum_Inhibin Alpha (NP_002182.1), Hum_Inhibin β A (NP_002183.1), Hum_Inhibin β E (NP_113667.1), Hum_Inhibin β B (NP_002184.2)

***Mus musculus***

Mus_BMP2A (P21274), Mus_BMP4 (P21275), Mus_BMP6 (P20722), Mus_BMP7 (P23359), Mus_BMP5 (NP_031581), Mus_BMP3 (Q8BHE5), Mus_Nodal (P43021), Mus_TGFβ1 (P04202), Mus_TGFβ3 (P17125), Mus_GDF8 (O08689), Mus_Inhibin Alpha (AAH56627), Mus_GDF9 (NP_032136), Mus_BMP8a (P34821), Mus_BMP8b (NP_031585), Mus_BMP9/GDF2 (Q9WV56), Mus_BMP10 (Q9R229), Mus_GDF3 (NP_032134), Mus_GDF1 (AAH79555), Mus_GDF5/BMP14 (P43027), Mus_GDF6/BMP13 (P43028), Mus_GDF7/BMP12 (NP_038555), Mus_GDF9b/BMP15 (Q9Z0L4), Mus_GDF10/BMP3b (NP_665684), Mus_GDF11/BMP11 (Q9Z1W4), Mus_GDF15 (AAH67248), Mus_Lefty A (BAA12121.1), Mus_Lefty B (NP_796073), Mus_Inhibin β B (NP_032407.1), Mus_Inhibin β C (NP_034695.1), Mus_Inhibin β A (EDL32727), Mus_Inhibin β E (NP_032408.2)

***Gallus gallus***

Gal_ADMP (NP_990153), Gal_BMP9 (P34822), Gal_TGFβ2 (P30371), Gal_MSTN (O42220), Gal_TGFβ3 (P16047)

***Xenopus laevis***

Xl_BMP2A (P25703), Xl_BMP4 (P30885), Xl_ADMP (AAC59736), Xl_BMP3b (Q7T2X6), Xl_BMP3 (Q7T2X7), Xl_BMP7 (AAT72008), Xnr5 (BAB18971), Xnr2 (AAA97393), Xl_GDF3 (AAH73508), Xl_GDF5 (AAT99303), Xl_Vg1 (AAH90232)

***Danio rerio***

Dan_ADMP (NP_571951), Dan_BMP15 (NP_001018320), Dan_BMP11 (AAN03678),

Dan_BMP5 (AAH54647), Dan_BMP4 (AAC60285), Dan_BMP2b (BAA24406), Dan_Lefty1 (NP_571035), Dan_Sqt (AAC34360), Dan_Cyc (AAC34361), Dan_MSTN (O42222), Dan_MSTN2 (AAT95431), Dan_ActivinBa (AAH66402 ), Dan_BMP7a(NP_571396), Dan_Vg1 (NP_571023)

***Takifugu rubripes***

Fugu_MSTN1 (NP_001027843), Fugu_MSTN2 (NP_001027844)

Cephalochordata

***Branchiostoma belcheri***

Amp_Nodal (BAC82629)

***Branchistoma floridae***

Bf_BMP2/4 (XP_002596858), Bf_BMP5/8 (XP_002588013), Bf_TGFβ (AEE90023), Bf_nodal (AAL99367), Bf_Univin (XP_002589220), Bf_Myostatin-like (XP_002603909), Bf_Myostatin (XP_002599461), Bf_GDF6/BMP13 (XP_002602867), Bf_ADMP (XP_002604737), Bf_Lefty (XP_002589231) ,Bf_inhibin_alpha (XP_035689281.1)

Urochordata

***Ciona intestinalis***

Ci_ADMP (BAE06303), Ci_TGFβ Lig (BAE06534)

Hemichordata

***Saccoglossus kowalevskii***

Sk_Activin (NP_001161496), Sk_BMP10 (XP_002735397),Sk_BMP2/4 (NP_001158387), SK_BMP3 (XP_002735398), Sk_BMP5/8 (NP_001158388), Sk_ADMP (NP_001158394), Sk_ADMP2 (NP_001161498), SK_GDF8/11 partial (XP_002734819), Sk_Lefty (NP_001164679), Sk_Myostatin-like/GDF2 (NP_001164699), Sk_Nodal A (ACY92597), Sk_Nodal B (ACY92598), Sk_Nodal C (NP_001164721), Sk_TGFβ (NP_001171727), Sk_Univin (XP_002732397)

Echinodermata

***Sea urchin***

Sp_Activin (GLEAN3_07004), Sp_Univin (P48970), Pl_BMP2/4 (DQ536194.1), Sp_MSTNA (GLEAN3_17647/XP_789990), Sp_BMP3 (GLEAN3_07822/XP_786367), Sp_Nodal (GLEAN3_11064/XM_774841/XM_796712), Pl_Nodal (AAS00534), Sp_TGFβ (GLEAN3_03835/XP_793246), Sp_BMP2/4 (NP_001116977), Sp_BMP5/8 (GLEAN3_12786/P48969), Sp_MSTNB (GLEAN3_02795), Sp_Lefty (GLEAN3_09911/XP_782698), Pl_Lefty (AAS00535), Sp_Maverick (GLEAN3_18248),

Pl_Univin (ABG00200), Pl_Maverick/GDF2 (AIF71192), Sp_MSTNA (GLEAN3_17647/XP_789990), Sp_BMP5/8 (GLEAN3_12786/P48969), Sp_MSTNC (GLEAN3_22079/XP_788027), Lv_Nodal (AAY41193), Pl_ActivinB (ACZ60068) Pl_ADMP1  (KP968256.1)

Et_Panda (***Eucidaris tribuloides*** )(Scaffold14736)

Et_ BMP10-like _partial: Scaffold34781

**Starfish**

***Patiria miniata*** BMP10 (000224) (XP_038062105.1)

***Acanthaster planci*** BMP10-like: >XP_022079545.1

**Sea cucumber**

***Aposticus japonicus*** BMP5/8-like (PIK57098)

**Ophiurids**

***Ophiotrix spiculata***_Panda-like

Mollusca

***Crassostrea gigas***

Crass_GDF3 (CAD67715), Crass_GDF2 (CAD67714),

***Lotia gigantea***

Lg_admp: jgi|Lotgi1|110168|

***Pecten maximus***

pecten Panda-like: XP_033738705.1

Annelida

***Platynereis dumerilii***

Plat_Dpp (CAJ38807),

Arthropoda

***Drosophila melanogaster***

Droso_Activin (O61643), Droso_myoglianin (NP_726604), Droso_Gbb (P27091), Droso_Scw (P54631), Droso_Dpp (P07713), Droso_Maverick (NP_524626), Droso_Dawdle (AAF51204)

Cnidaria

***Hydra vulgaris***

Hyd_BMP5/8 (AAS01764)

***Nematostella vectensis***

Nv_Activin (ABF61781), Nv_MSTNA (AGL96595), Nv_GDF5 (AAS77520), Nv_Dpp (AAR27580), Nv_BMP5/8 (ABC88372), Nv_ADMP (AFP87424)
