# Supplementary material for: Maternal TGF-β ligand Panda breaks the radial symmetry of the sea urchin embryo by antagonizing the Nodal type II receptor ACVRII
Source: PLoS Biol. 2024 Jun 24;22(6):e3002701. doi: 10.1371/journal.pbio.3002701 (PMC11239237; doi:10.1371/journal.pbio.3002701)
Supplement: S1 Raw Images — (PDF) [file pbio.3002701.s010.pdf]

FIGURE 4A

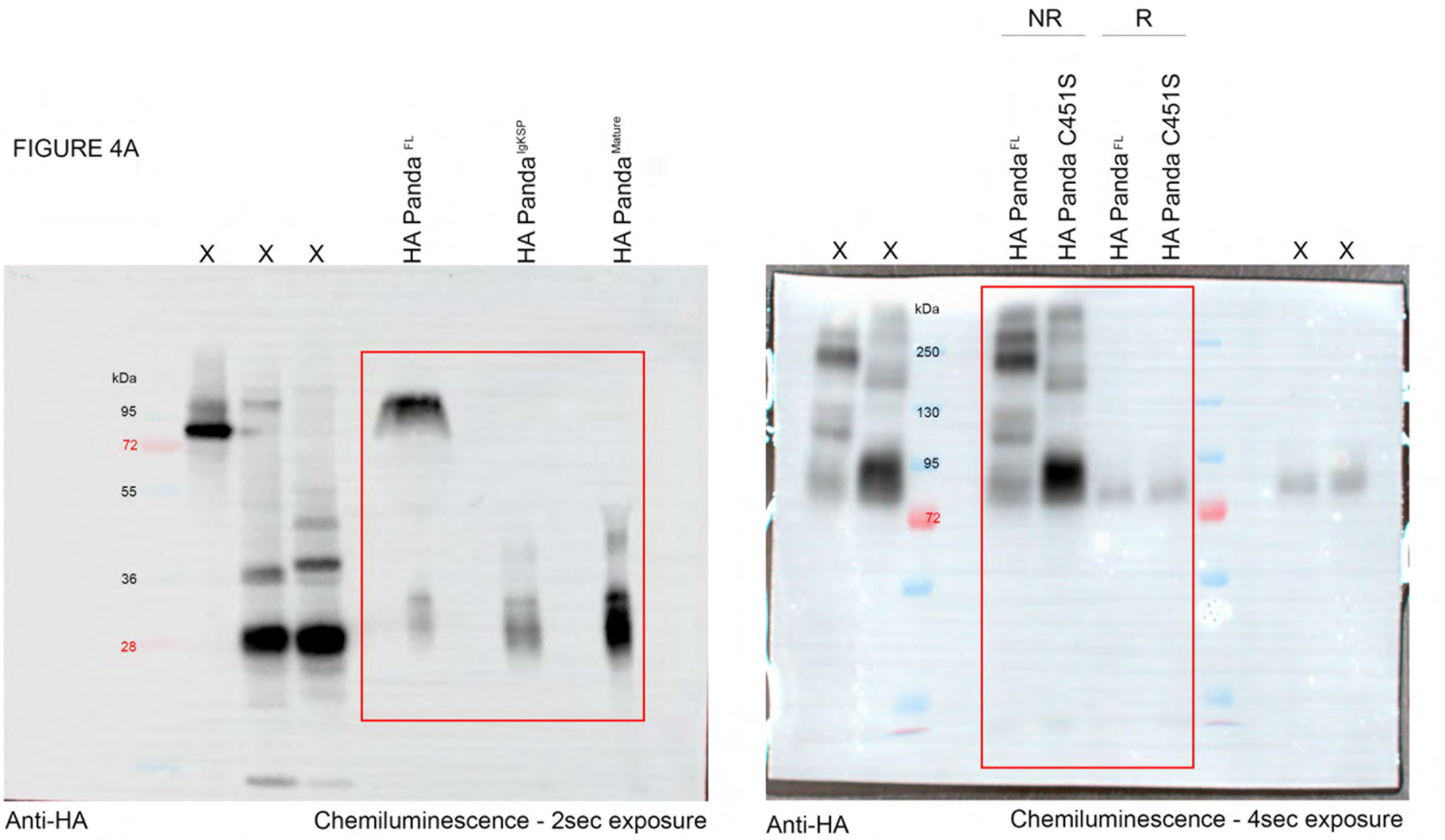
X
X
X
HA Panda FL
HA Panda IgKSP
HA Panda Mature
kDa
95
72
55
36
28
Anti-HA
Chemiluminescence - 2sec exposure
NR
R
X
X
HA Panda FL
HA Panda C451S
HA Panda FL
HA Panda C451S
X
X
kDa
250
130
95
72
Anti-HA
Chemiluminescence - 4sec exposure

FIGURE 4B Part1

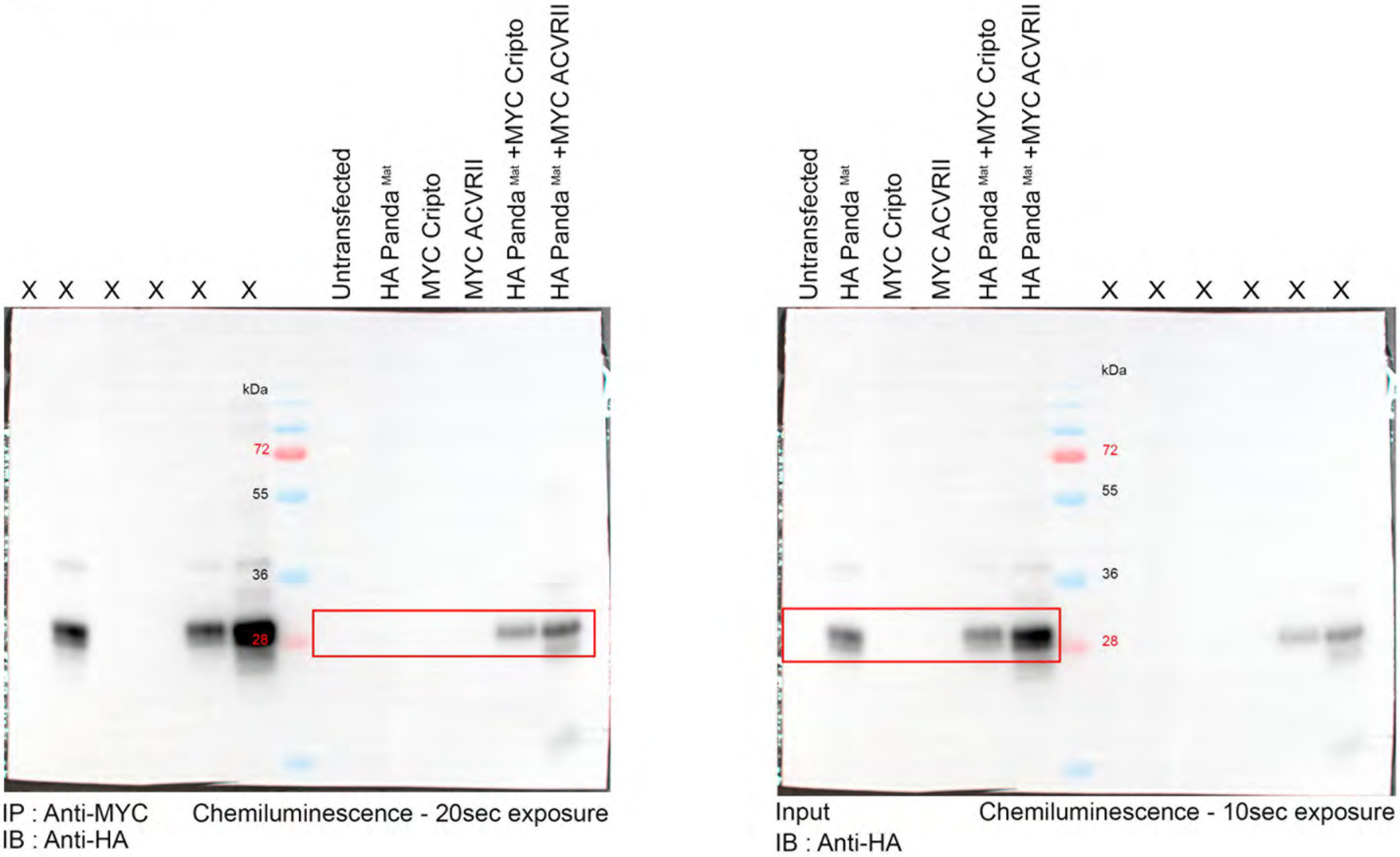

FIGURE 4B Part2

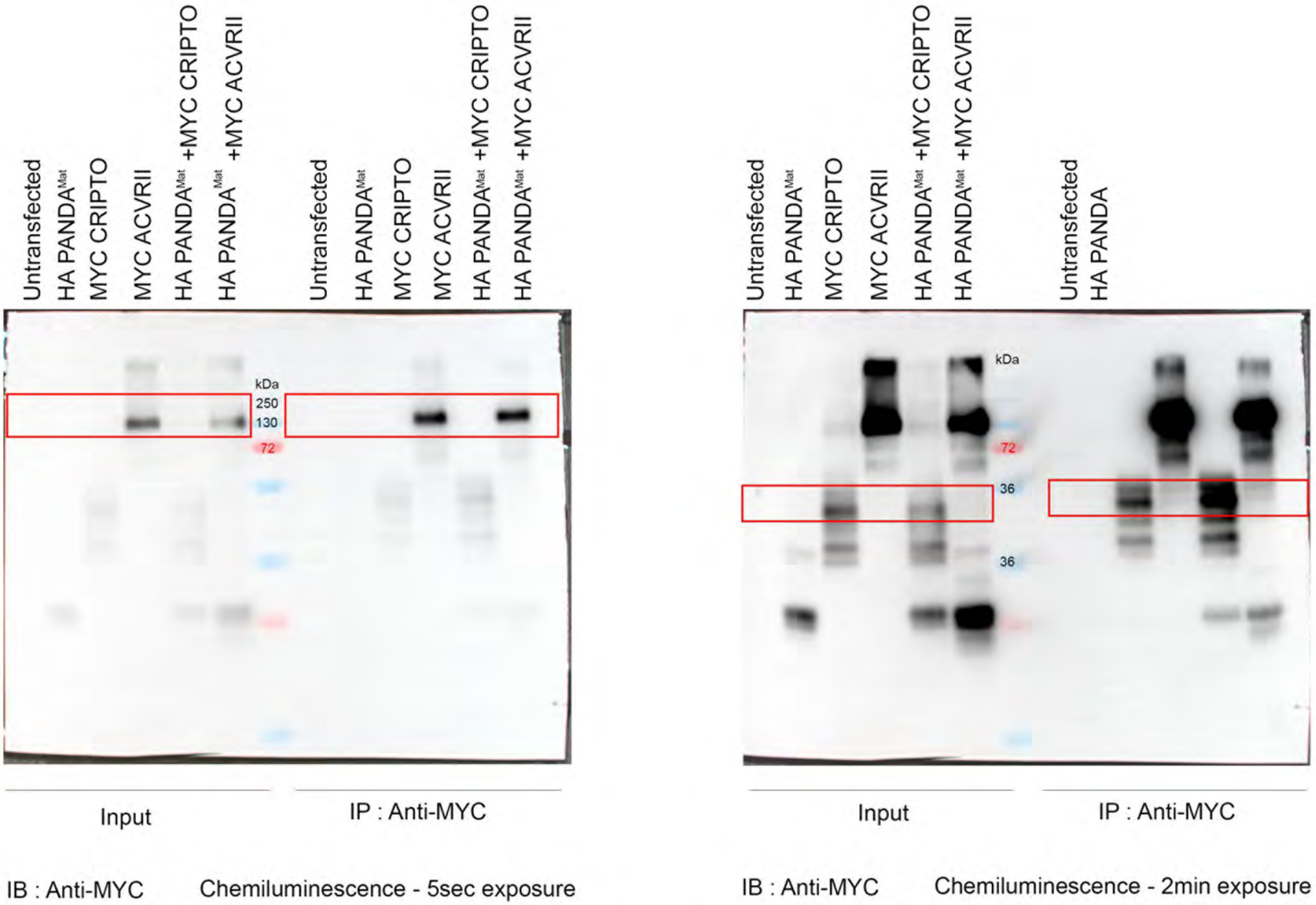

FIGURE 4C Part 1

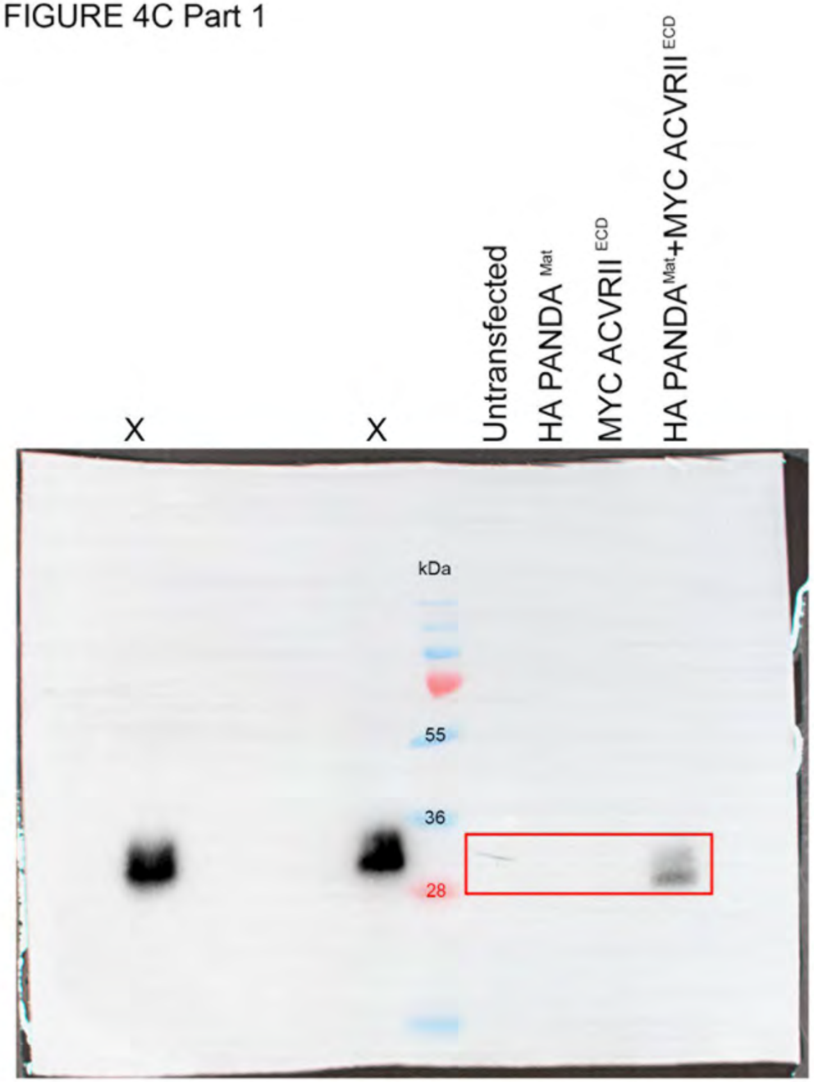

IP : Anti-MYC Chemiluminescence - 1min30 exposure
IB : Anti-HA

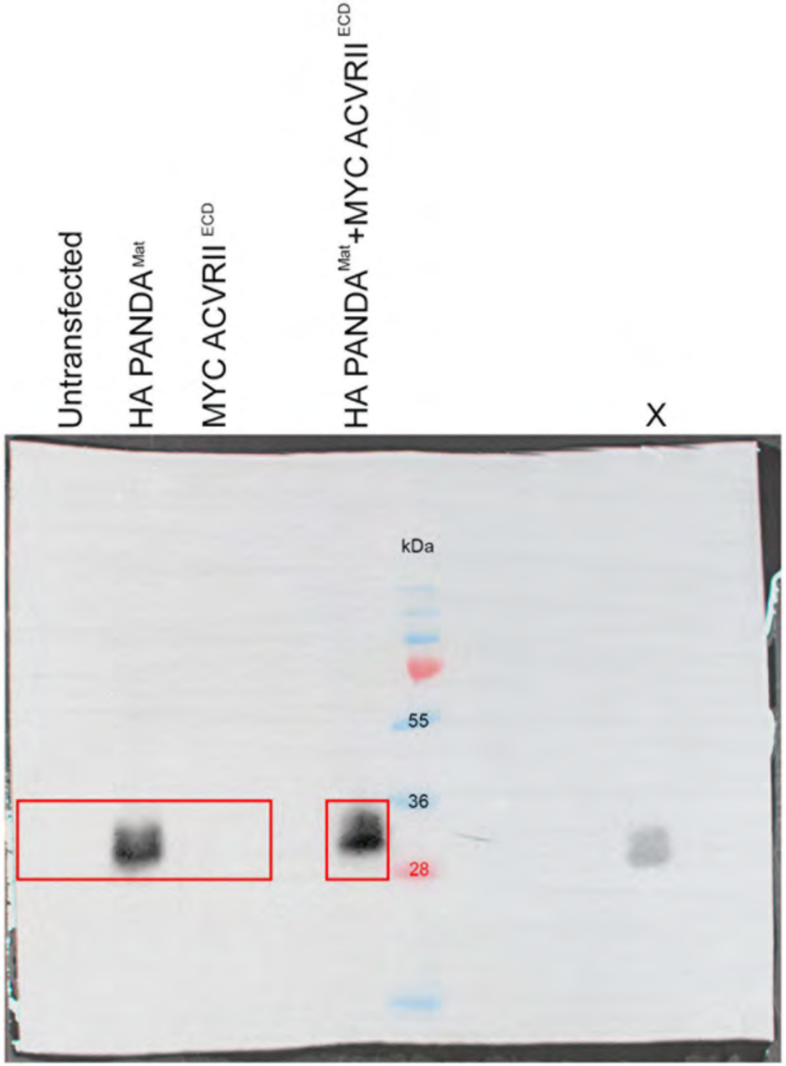

Input Chemiluminescence - 15sec exposure
IB : Anti-HA

FIGURE 4C Part 2

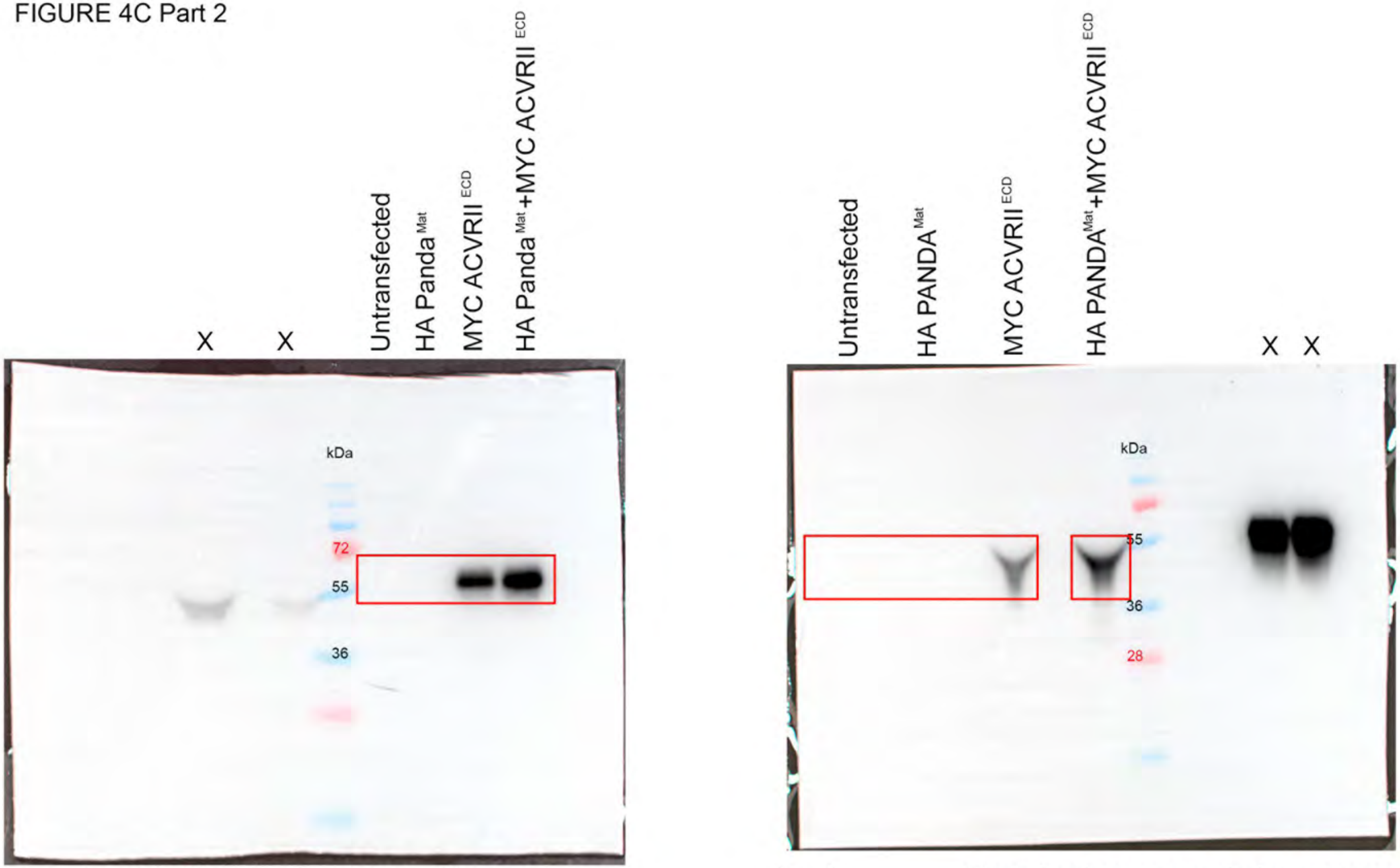

IP : Anti-MYC Chemiluminescence - 10 sec exposure
IB : Anti-MYC

Input Chemiluminescence - 1min exposure
IB : Anti-MYC

FIGURE 4D Part 1

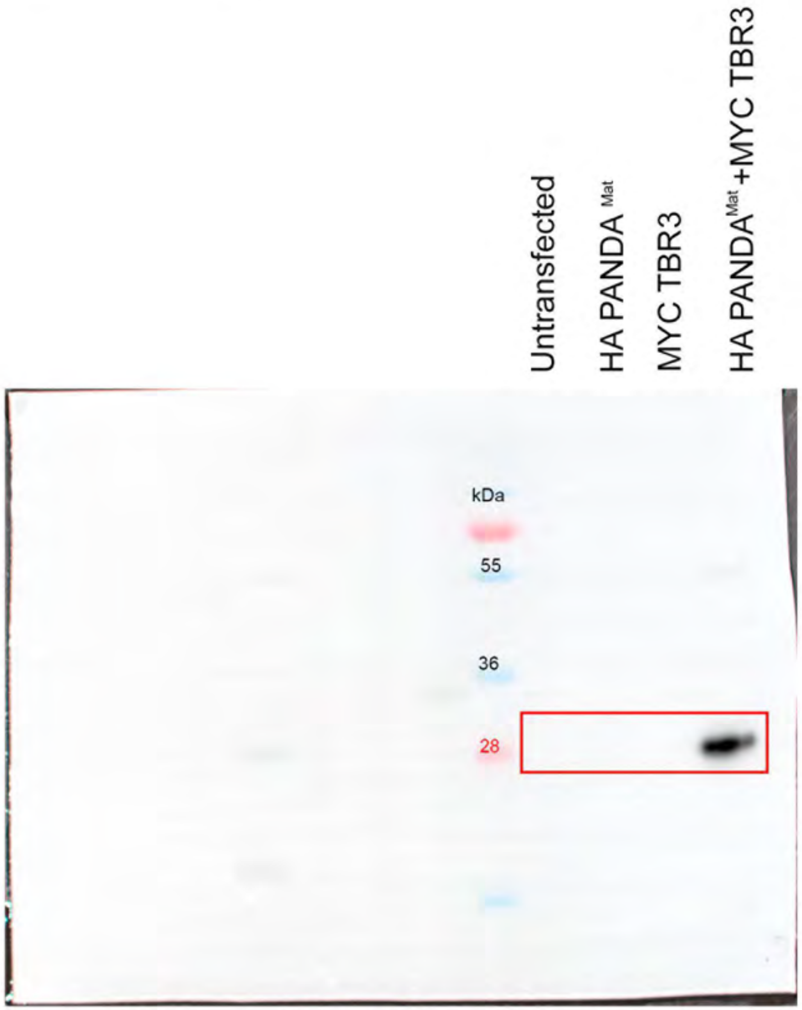

IP : Anti-MYC
IB : Anti-HA

Chemiluminescence - 5sec exposure

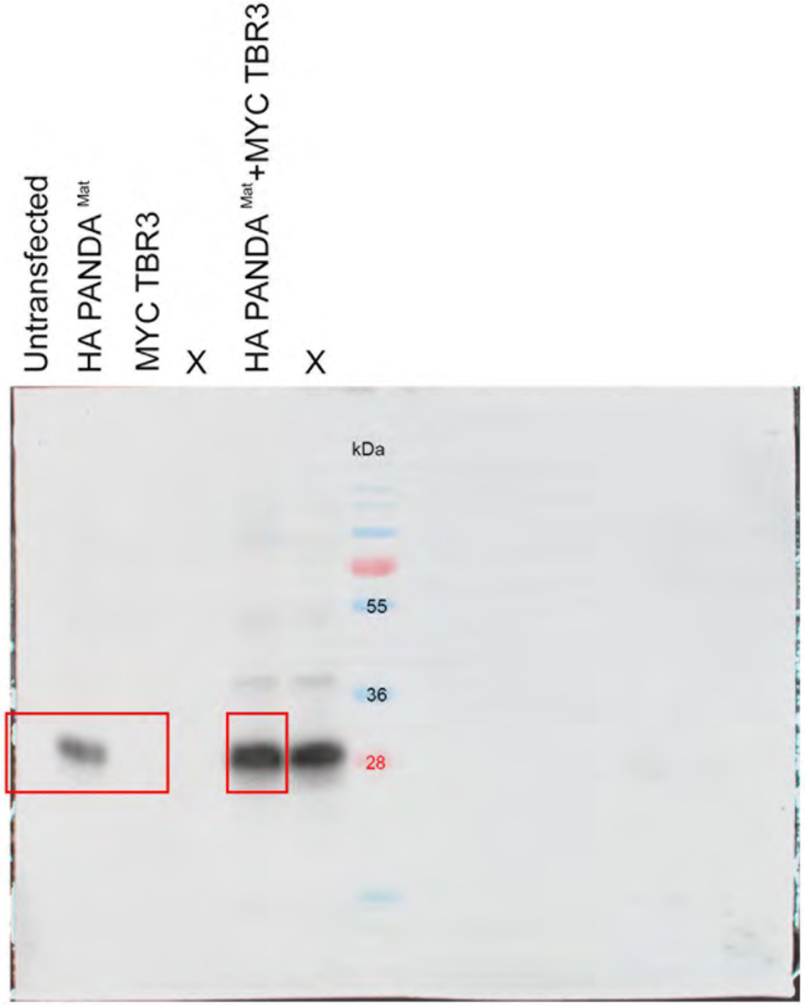

Input
IB : Anti-HA

Chemiluminescence - 1min exposure

FIGURE 4D Part 2

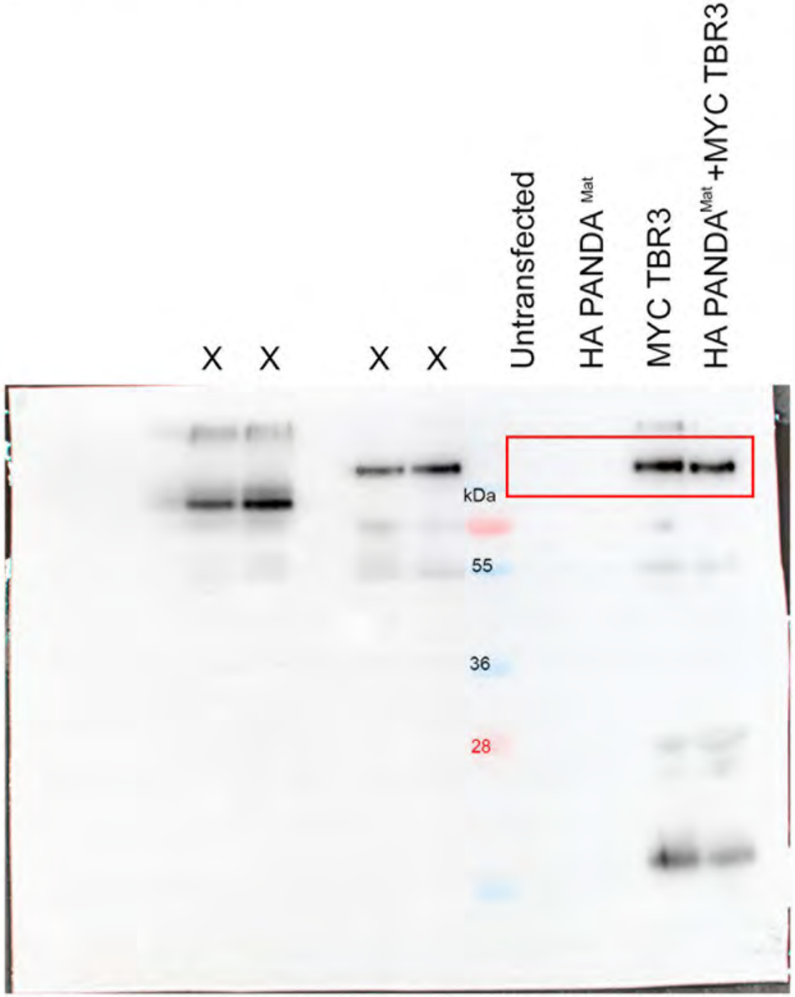

IP : Anti-MYC
IB : Anti-MYC
Chemiluminescence - 5sec exposure

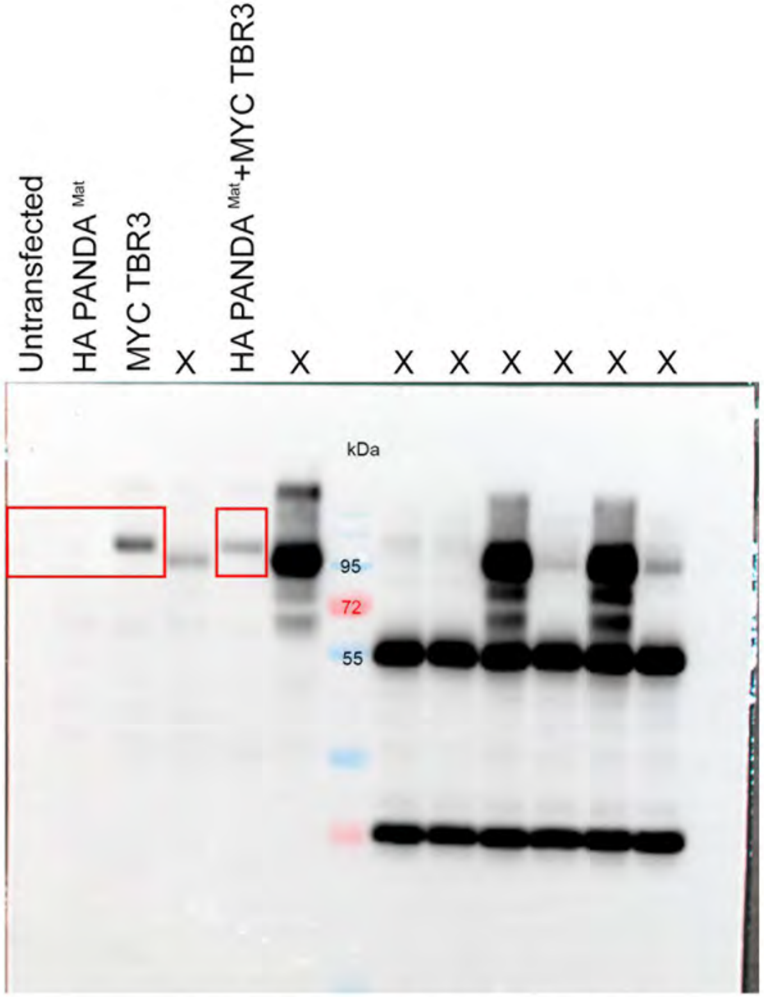

Input
IB : Anti-MYC
Chemiluminescence - 1min exposure

FIGURE 4E Part 1

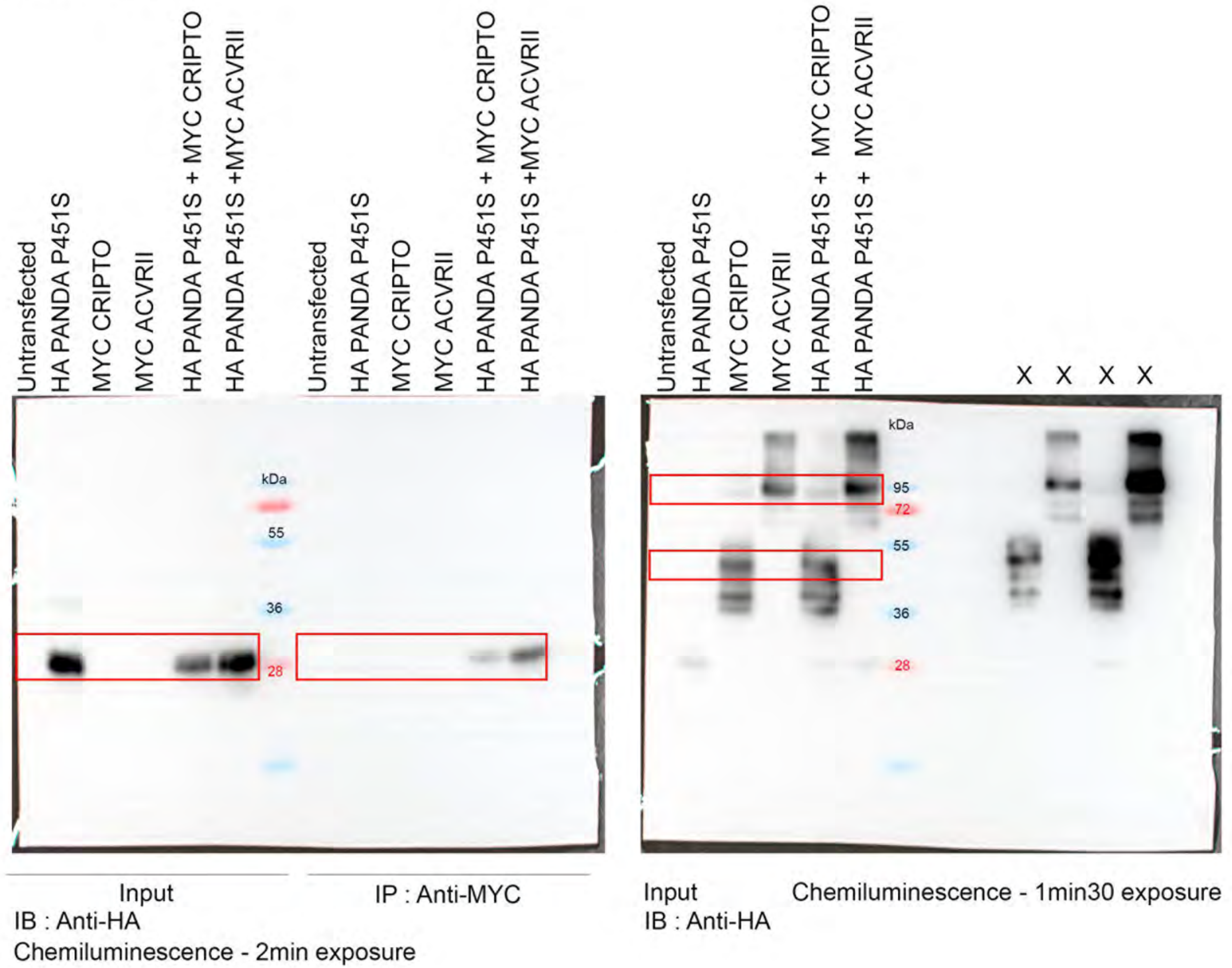

FIGURE 4E Part 2

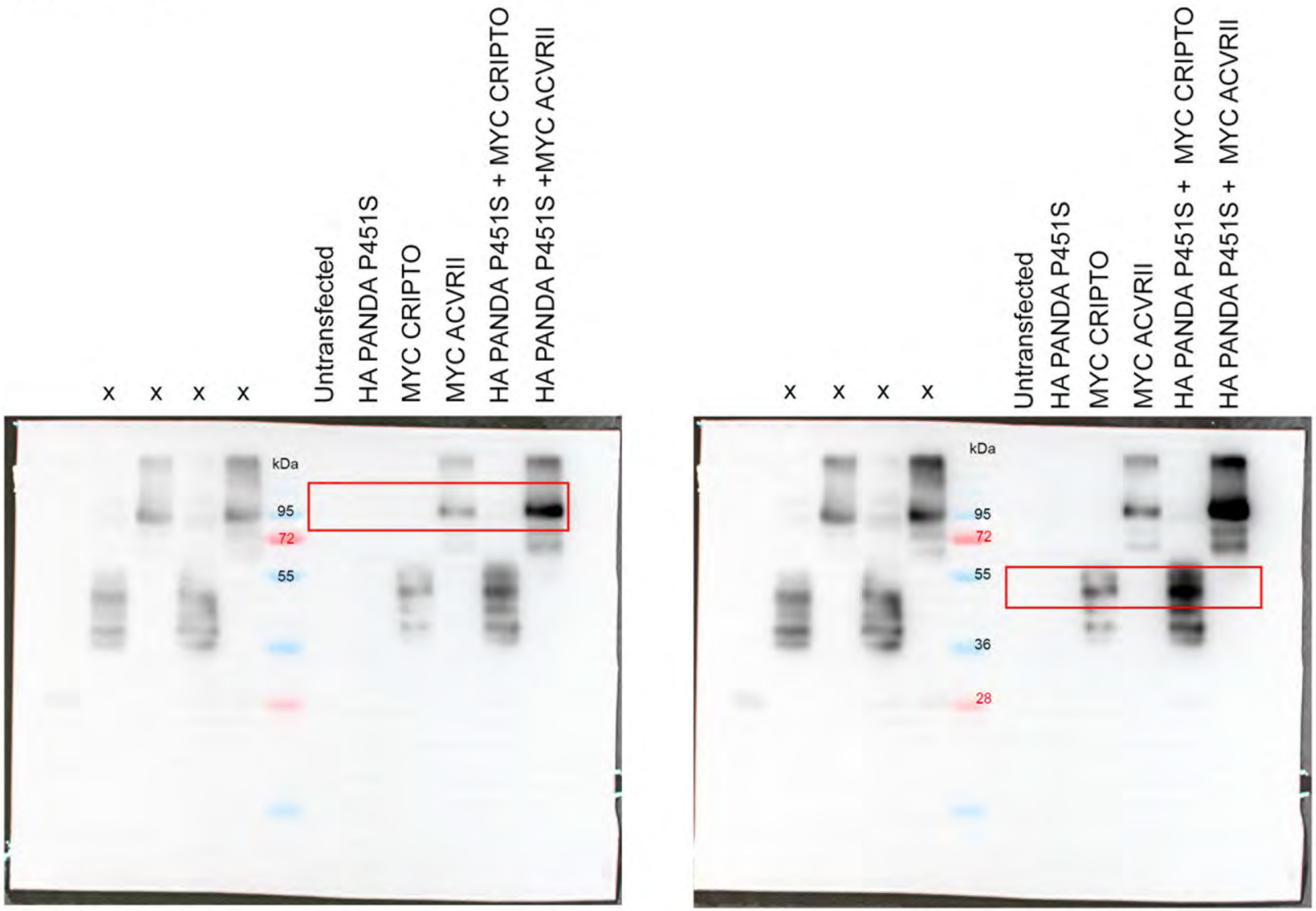

IP : Anti-MYC
IB : Anti-MYC
Chemiluminescence - 10sec exposure

Input
IB : Anti-MYC
Chemiluminescence - 1min10 exposure

FIGURE 3S part1

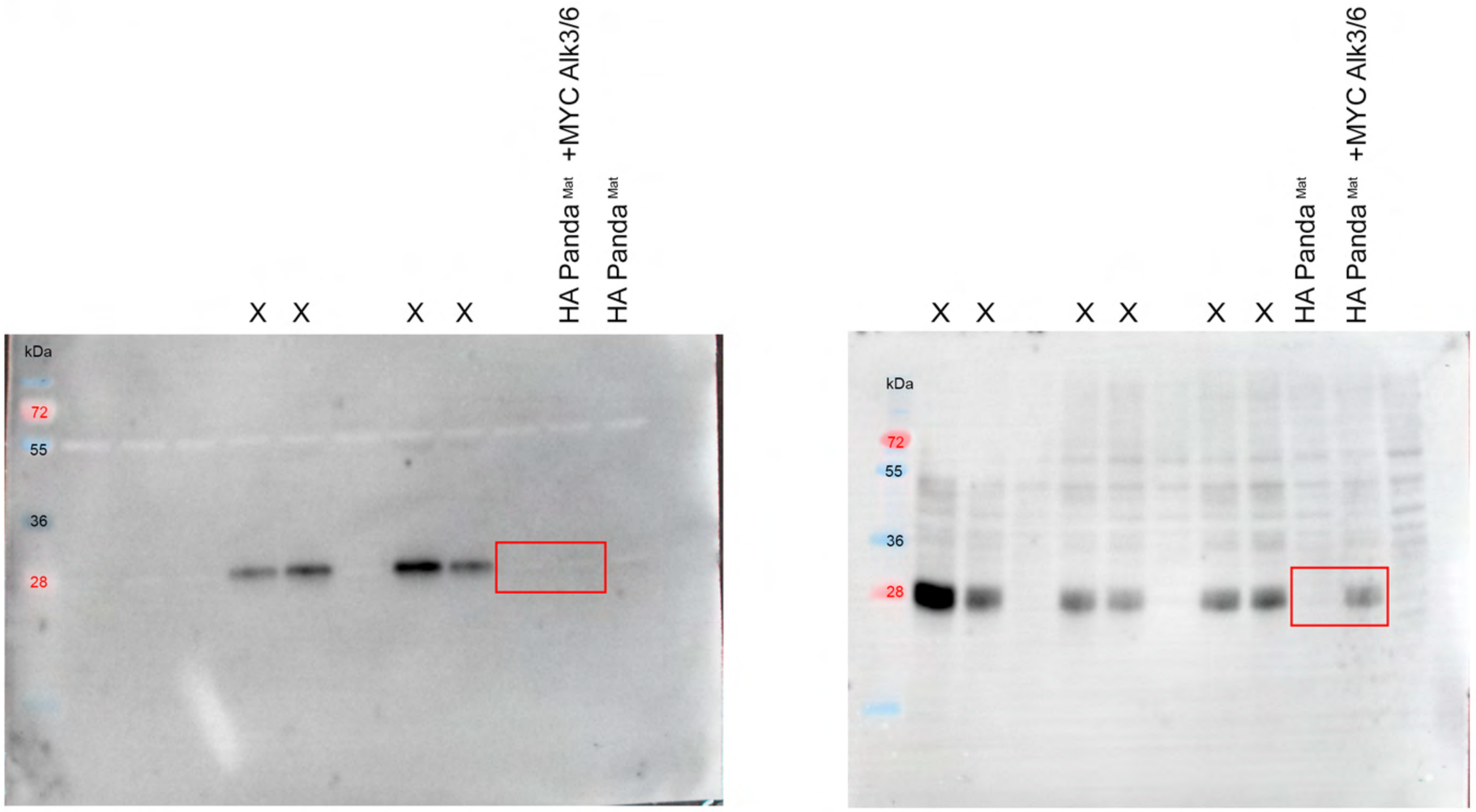

IP : Anti-MYC
IB : Anti-HA

Chemiluminescence - 1min40 exposure

Input
IB : Anti-HA

Chemiluminescence - 3min exposure

FIGURE 3S part 2

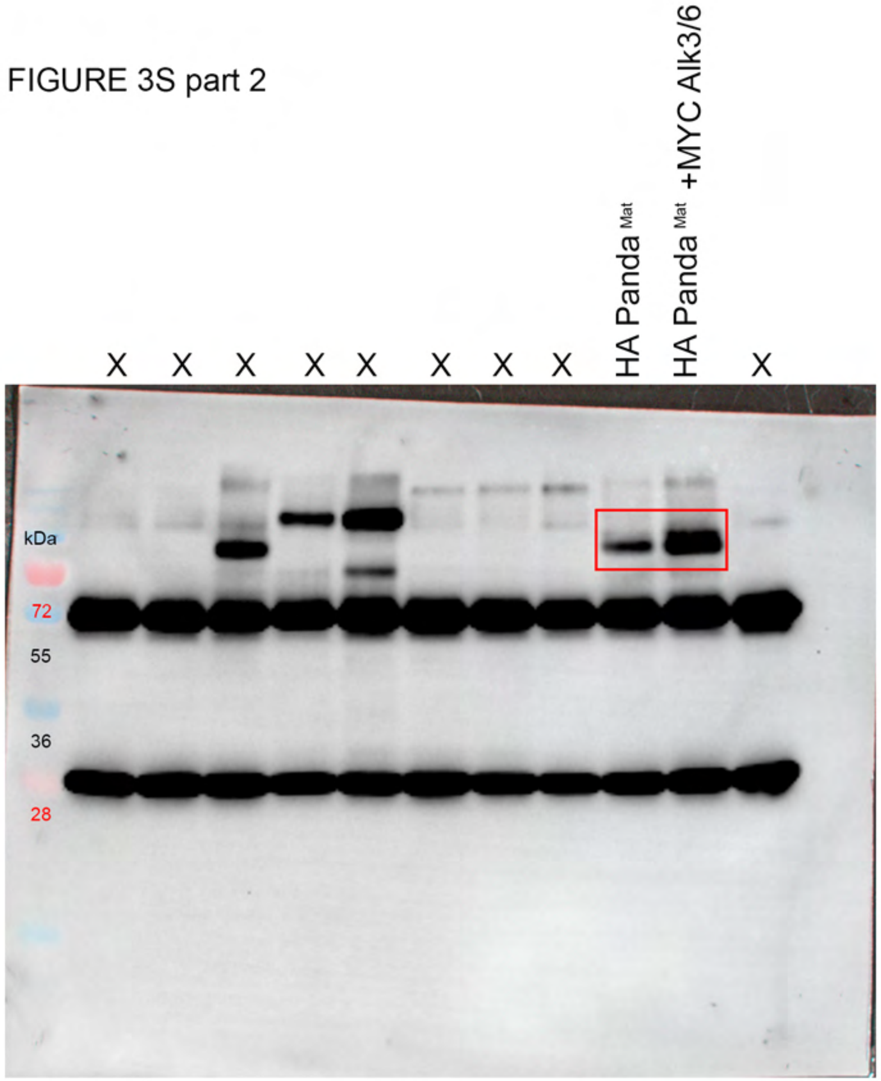

IP : Anti-MYC
IB : Anti-MYC

Chemiluminescence - 30sec exposure

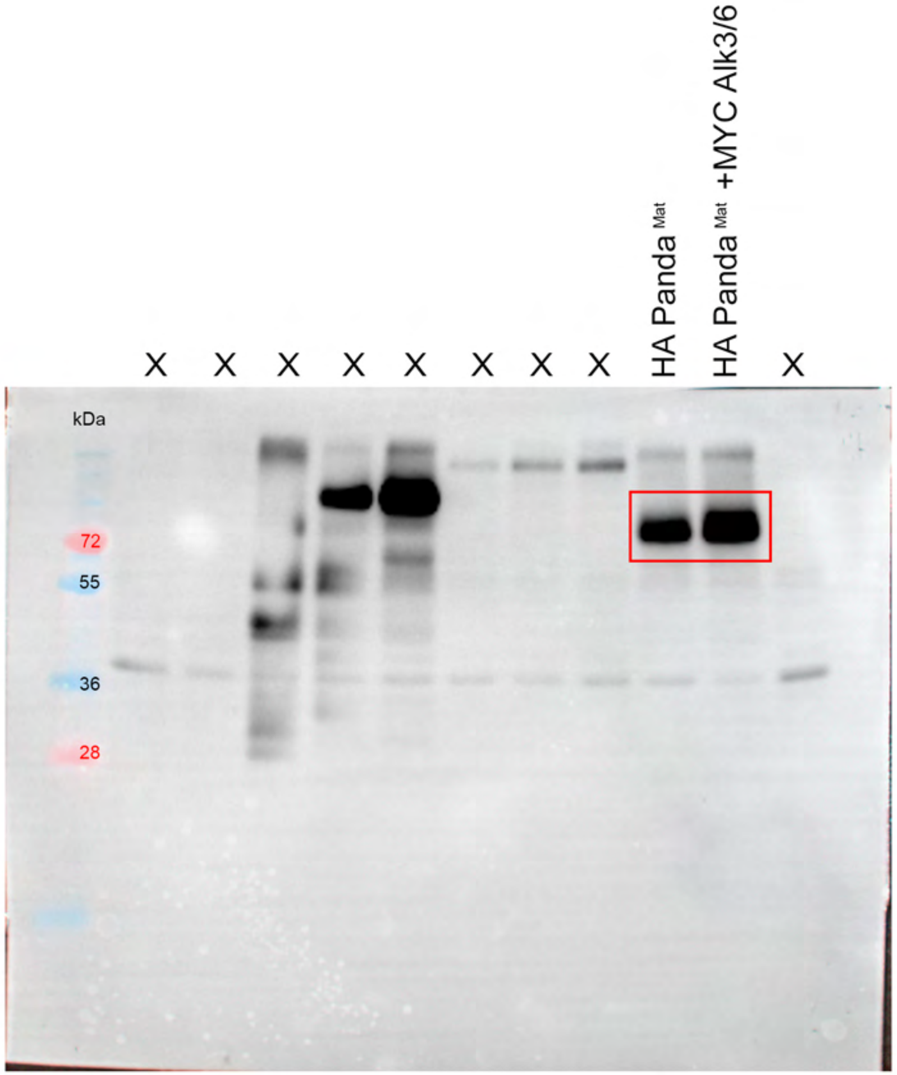

Input
IB : Anti-MYC

Chemiluminescence - 2min exposure
