# Supplementary material for: Maternal TGF-β ligand Panda breaks the radial symmetry of the sea urchin embryo by antagonizing the Nodal type II receptor ACVRII
Source: PLoS Biol. 2024 Jun 24;22(6):e3002701. doi: 10.1371/journal.pbio.3002701 (PMC11239237; doi:10.1371/journal.pbio.3002701)
Supplement: Attachment — Submitted filename: Point by point Response to the Reviewers.docx [file pbio.3002701.s011.docx]

*Reviewers' comments*

*Rev. 1:*

*1. All the evidence presented is consistent with Panda acting as an ACVR2 antagonist, except one: Panda overexpression within 1-celled embryos does not phenocopy ACVR2 morphants and in fact has almost no phenotype at all. Why, if Panda blocks the activity of overexpressed ACVR2, does it not also block endogenous ACVR2 function? And how does Panda overexpression affect Nodal expression?*

The reviewer points that although the data point to Panda acting as an antagonist of ACVRII, surprisingly, overexpression of Panda into one-cell zygotes does not cause any morphological phenotype. The reviewer therefore asks how does Panda overexpression affect nodal expression? We agree with the reviewer regarding the surprising lack of effect of Panda overexpression into the egg. This is really surprising and particularly in light of our results showing that Panda antagonizes overexpressed ACVRII. To address this question, we have overexpressed Panda mRNA at high concentration (1500 microgram/ml) into the egg and performed nodal in situ at blastula stage. For the first time, we were able to show that, although panda overexpression does not abolish nodal expression, it does significantly reduces the level of endogenous nodal transcripts. This effect is however, transient since these embryos will regulate and develp into normally patterned pluteus larvae. Therefore Panda behaves as an antagonist of Nodal signalling but is appears to be a considerably less potent inhibitor compared to for example lefty.

*2. The authors show that ACVR2 activates ectopic pSmad1/5/8 even in the absence of BMP5/8 and conclude that, because no other BMPs are expressed at this time, that this activity of ACVR2 must be ligand independent. However, they also note that BMP2/4 is induced by Nodal at later stages of development. Isn't it possible then that ACVR2 overexpression accelerates induction of BMP2/4 by Nodal signaling and the increase in pSmad1/5/8 is dependent on this ligand? To help distinguish between these possibilities, the authors should examine expression of both BMP2/4 and Nodal upon ACVR2 overexpression.*

The reviewer is correct to point that formally, we cannot exclude that ACVRII overexpression may causes premature ectopic expression of nodal which in turn would induce *bmp2*/4 expression causing ectopic phospho Smad1/5/8 activation.

To address this question, we have overexpressed ACVRII and performed nodal and bmp2/4 in situs at early blastula stage as requested. The results show that *acvr2* overexpression does not cause ectopic expression of *bmp2/4* at VEB stage. We have therefore added the following sentence to the main text: "Finally, although embryos injected with *acvrII mRNA* showed ectopic activation and nuclearization of pSmad1/5/8 at the VEB stage, they did not show ectopic expression of *nodal* or *bmp2/4* indicating that the ectopic pSmad1/5/8 signalling observed following overexpression of *acvr2* is not caused by a premature expression of *nodal* and/or *bmp2/*4".

*3. Here, the authors find that the ability of Panda to promote dorsal fates upon injection into a single blastomere does not depend on BMP receptors. However, these findings are opposite to those reported by this same group in a previous study (Haillot et al, 2015). What explains this discrepancy?*

The reviewer is correct to point that the dependency of Panda on BMP receptors described in this paper is different from that described in the original study. We think that the discrepancy between the two studies is due to the too small statistics associated to this experiment in the first study. The first conclusion (Panda is dependent on BMP receptors to orient the axis) was based on a single experiment with only 23 embryos while the second conclusion (Panda is independent on BMP receptors to orient the axis) relies on 3 different experiments with a total number of embryos analyzed of 176. In these experiments, the ability of Panda mRNA to orient the D/V axis was100%, 100% and 95%. We are therefore much more confident by these last experiments

*4. The authors speculate that although ACVR2 can activate BMP upon overexpression and Panda can block this activity, Panda acts predominantly as a Nodal inhibitor in vivo. To confirm this experimentally, they could similarly test the ability of Panda to block pSmad1/5/8 activation by BMP ligands or Alk1/2 / Alk3/6 overexpression. If Panda cannot counteract BMP signaling through its canonical players, this would provide further support for this claim.*

Following the idea of the reviewer, we have overexpressed Panda alone or in the presence of an activated BMP type I receptor (Alk1/2 QD) into the egg and at blastula stage we performed pSmad1/5/8 immunostaining (Fig S1). The results show that overexpression of panda does not antagonize signalling from Alk1/2QD. We have added the following sentence to incorporate this piece of data into the manuscript.

"In contrast, overexpression of *panda* did not antagonize pSMAD1/5/8 signalling induced by overexpression of the constitutively active BMP receptor Alk1/2QD. This suggests that in vivo, Panda may work predominantly as a Nodal signalling inhibitor"

*5. The authors further speculate that Panda may antagonize Nodal signaling by forming heterodimers with Nodal ligands and claim in the discussion to have preliminary data demonstrating a direct interaction between these ligands. These data should be included in this study.*

During the revision period, we have extensively tried to get additional evidence that Panda and Nodal heterodimerize.

Unfortunately, we have not been able to further support the idea that Panda heterodimerises with Nodal by doing co-IP. For unknown reasons, we had very low levels of expression when we co-expressed panda and Nodal. Since these technical difficulties prevented us to provide additional support for our hypothesis we have removed from the paper the mention about preliminary data showing that Panda and Nodal coIP. We now only refer to this idea as an hypothesis that remains to be validated.

*6. Although embryo numbers are provided in the text and figures for some experiments, the number of embryos analyzed and independent experimental trials should be provided for all experiments, including co-IPs.*

We now provide a file indicating the number of embryos analyzed and of independent experiments, including coIP.

*Minor point:*

*1. The authors state multiple times that ACVR2 is a limiting factor for Nodal signaling within the embryo. But if that were the case, why does its overexpression instead promote BMP signaling? To me, this suggests that Nodal is limiting and therefore, excess ACVR2 that is unoccupied by Nodal is then diverted to BMP.*

The reviewer argues that since overexpression of ACVRII promotes BMP signalling, Nodal signalling, not ACVRII, is limiting and therefore *, excess ACVR2 that is unoccupied by Nodal is then diverted to BMP.*

We understand the point raised by the reviewer. However, it is important to keep in mind that ACVRII is a type II receptor for both Nodal and BMP signals. Furthermore, ACVRII is a constitutively active kinase. Our interpretation regarding the early activation of BMP signalling following overexpression of ACVRII is that overexpressed ACVRII associates with and phosphorylates both the Nodal receptor Alk4/5/7 and the type I receptors Alk1/2 and Alk3/6 resulting in the precocious activation of both Nodal and BMP signalling.

We favor the idea that ACVRII is a limiting factor for Nodal signalling based on the loss-of- function experiment with the morpholino knock-down. This experiment shows that reducing the level of ACVRII causes first a Nodal loss-of-function phenotype. Therefore, in the unperturbed embryo, ACVRII appears to be limiting for Nodal signalling.

*Rev. 2:*

*This manuscript provides an update to the function of Panda, a TGF-ß family member, that is a maternally provided transcript in the sea urchin egg. Experiments here reinforce expand what is known about how Panda functions. Previously, based on indirect evidence, it was proposed that Panda was crucial for the establishment of the dorsal-ventral axis by setting up an early asymmetry in the function of Nodal. The proposal was that Panda acted through ~~inhibition~~ activation of two BMP receptors, Alk3/6 and Alk1/2, to restrict nodal expression to the ventral side, thereby establishing D-V symmetry. These experiments were indirect and did not fully establish that it was binding of Panda to the two BMP receptors, but was presented as a model of that possibility. Here, after further investigation, the Lepage group reports that the idea of Panda being involved in establishing D-V asymmetry by restricting Nodal expression to the ventral ectoderm is reinforced, but through a different receptor,*

*ACVRII, a type II TGF-B receptor. Rather than indirectly proposing this as was done in the previous study, this group performed a number of biochemical studies to show that the binding of Panda was due to a direct interaction with ACVRII by way of a sequence that included a proline in the binding site, a finding that puts Panda in a class of ligands that includes Lefty, another Nodal antagonist. As such the updated information moves forward an important understanding of how the D-V axis in sea urchins is established to set up Nodal asymmetrical signaling. Further, although the data is not overwhelming, there is some evidence that maternal Panda is distributed asymmetrically in the egg, perhaps setting up the sequence of events that leads to the D-V asymmetry.*

*Below are editorial suggestions that this reviewer believes will improve the report. The one concern I have beyond these is the discussion. Much of the second half of the discussion is speculation on details of what might be going on in as yet unanswered questions. I recommend that this section be much reduced. The data in the Results section strongly supports the conclusions they reach. Yes, there are details remaining to be established, but speculation on what those details might yield are, in my opinion, unnecessary here. I suspect this group will do the experiments and it will be appropriate to learn from them when the speculation becomes information.*

The reviewer suggested to reduce significantly the Discussion section and particularly the second part. We have followed this advice. The Discussion, which was previously 4347 words has been shortened to 3448 words.

*1. Ln 125-139. You cite two of your papers indicating that Nodal is expressed slightly before Lefty. That makes sense since Nodal is upstream of Lefty, but those two papers do not provide evidence to support the statement. Instead, they indicate, before you did a higher sensitivity in situ, that both genes are first expressed at the 60-cell stage.*

The reviewer points that the two references that we cite do not provide evidence that support the idea that Lefty is expressed after nodal.

We agree with the comment of the reviewer. This is why in addition to the Duboc et al. 2004 and Saudemont et al. 2010 papers, which show expression of Nodal at 60-cell stage, we have added one reference describing the expression of Lefty (Duboc et al. 2008 Dev. Biol.), that shows a Northern blot and in situ hybridizations for Lefty with no expression at 60-cell-stage and expression starting at the early blastula stage.

*2. Since most of your earlier papers referred to Alk4/5/7 as the Nodal receptor, it will be value on line 205-206 to clarify the relationship between that earlier descriptive and your current descriptive of ACVRII - and be consistent in the use of that or ACVR2. OK—I now come across your explanation of the relationship of all these molecules - so on line 205 you might add (see below) so the reader will put off his/her confusion - as I was confused at that point.*

Apparently, the reviewer was confused by the first mention of ACVRII as being the receptor for Nodal while our previous papers only mentioned Alk4/5/7 as being the Nodal receptor. However, since we mention ACVRII as being the type II Nodal receptor, we think that this ambiguity is removed.

*3. Line 257. You state that ACVRII overexpression was expected to activate Nodal signaling. Don't you mean you would expect it to augment Nodal signaling since Nodal is there?*

We agree with the reviewer and prefer the verbs "augment" or "promote" suggested by the reviewer compared to "activate" that was used in the text. We have therefore replaced "activate" by "promote" in the text line 257.

*4. On Figure 1C, ;The wild type 48 and 72h embryos are labeled lateral and vegetal, but they appear to be in the wrong position with the vegetal view in the lateral panel and the lateral view in the vegetal panel. Also, in Fig. 1 there should be a bar approximating the size of the embryo. It looks to me as though all the figures are about the same level of magnification so a single bar should suffice.*

We thank the reviewer for pointing us these errors. We have corrected the orientation of the embryos as suggested. We also included a magnification bar in the first panel of Fig.1A as requested.

*5. Line 310. The conclusion of this section is fine but it likely depends on a certain ratio of the two treatments. Is that the case? In other words, if you titre the Panda morpholino there is an expected series of outcomes with a titre much higher than the ACVRII overexpression eliminating all ACVRII activity. You seem to report the outcome that would be expected if Panda concentration erased the overexpression down to approximate the control level of expression.*

The reviewer argues that the rescue of the effect of ACVRII overexpression by Panda overexpression is likely to be concentration-dependent.

The reviewer is correct to point that the rescue of ACVRII overexpression by Panda mRNA is likely to be concentration dependent. We has injected different ratios of ACVRII/Panda 0.6, 0.75, 1.75 and the phenotype show a gradation with full rescue at 0.6, partial rescue at 0.75 and no rescue at 1.75

*6. Better stated on line 344: For this, acvrII mRNA along with a lineage tracer was injected into one blastomere of embryos at the two-cell stage.*

We thank the reviewer for this suggestion.

*7. Fig. 2b is confusing. The left panel shows the ventral side is the injected side. I suggest you put in outcome numbers on the figure. The middle panel shows what? And what are the numbers of that outcome? And the right panel is different from that but your figure legend and text don't indicate what it is. Are both outcomes of the ACVRII + panda injection? If so, include the numbers. But if so, how do you explain the far right image when you say Panda blocks the ability of ACVRII to impose a ventral identity? Fig. 2C provides the numbers but it would help if the 2B panel were better annotated perhaps by incorporating the perentages directly on the images with what I am assuming is Lateral and on the far right, ventral. Also, instead of saying the impact of ACVRII is inhibited, I would say it was cancelled.*

We have followed the suggestion of the reviewer and incorporated the percentages directly in the panels. We also adopted "cancelled" rather than "blocked line 396. "

*8. Figure 2D and E are excellent. And also the table is excellent in supporting the hypothesized function of Panda.*

*Rev. 3:*

*The current studies provide clear biochemical and phenotypic evidence that the mechanism by which the maternally expressed TGFß family member Panda regulates nodal signaling is like that of Lefty, by sequestering ACVRII. They also show that Panda binds to Betaglycan, raising the possibility that it may use betaglycan as a binding partner to increase binding affinity for ACVRII, similar to Inhibins. The studies are rigorous and clearly presented for the most part but there are a few issues that should be addressed to improve the manuscript:*

*Major recommendations:*

*1. It's important to know if the biologically active form of Panda is a monomer or dimer, particularly in consideration of the possibility that PandaP461S may function as a dominant mutant (see point 4 below). The authors should repeat the experiments shown in Panel 3A under reducing and non-reducing conditions.*

The reviewer points that it is important to know if Panda works as a monomer or as a dimer

and suggests that we repeat the experiment in Fig. 4A under reducing and non-reducing conditions. We have followed the reviewer suggestion. In addition, to further document the dimerisation state of Panda, we made a Panda mutant in which the cysteine required for dimerisation (cysteine 451) was replaced by a serine.

These experiments clearly show that under non-reducing conditions, Panda is present in the medium of transfected cells predominantly as high molecular weight precursor proteins of 160 and 250 kDa. In contrast, under reducing conditions or in the Panda P451S mutant, these high molecular weight bands disappear and are replaced by a predominant form of Panda with a MW of about 80 kDa. These results therefore strongly suggest that Panda is secreted as a dimer in the medium of transfected cells and that cysteine 451 of Panda is required for dimerisation.

*2. Does Panda co-IP with Type I nodal receptors? (ALK4,5,7)? This is more relevant than showing that it does not bind to Type I BMP receptors since the endogenous protein primarily affects nodal activity.*

We have tried to address the question of the interaction between Panda and Alk4/5/7 by doing co-IP. Unfortunately, for technical reasons, we have not been able to reach a conclusion regarding this question. The problem we encountered was that when co-expresed, the levels of Alk4/5/7 and Panda we very different making any conclusion difficult to draw.

*3. The authors generate a point mutant form of Panda in which a conserved Proline residue located within the ACVRII binding knuckle region in "inhibitory" TGFß ligands is substituted with a Ser that is conserved in agonists. Ectopic expression of PandaP461S induces ectopic nodal expression and local injection imposes a ventral identity. They conclude "Lines 546-547: Collectively, these results suggest that proline 461 of Panda is crucial for its function as an antagonist of Nodal signaling, and that conversion of the proline to a serine switches the activity of Panda from that of an antagonist of Nodal signaling to that of an agonist." I agree with the statement that this proline is crucial for antagonist activity, but the evidence that the mutation converts it from an antagonist to an agonist is not strong. An alternate interpretation is that the switch converts PandaP461S to a dominant mutant form that inhibits wild type Panda to indirectly upregulate nodal. Further*

*evidence that PandaP461S can directly activate ACVRII is required to support the conclusion that it has agonist activity. (e.g. demonstrating in a heterologous system that it can directly induce pSmad1 or pSmad2, or at least demonstrating that PandaP461S doesn't block the activity of WT panda when co-expressed).*

We thank the reviewer for this comment and we understand the concern of the reviewer. It is perfectly correct that the data regarding this mutant could be be due to this mutant working as a dominant negative form of Panda. To test this hypothesis, we have performed axis induction assays using either wt Panda or the Panda P461S mutant. When injected into one blastomere, panda P461S imposed a ventral identity to the clone of injected cells while injection of wt panda promoted dorsal fates. Co-injection of pandaP461S and wild type panda clearly promoted dorsal fates, exactly like co-injection of wt panda. Therefore, we conclude that panda P461S does not work as a dominant negative form. Furthermore, we found that mutation of Y426, a residue essential for type receptor binding in pandaP461S prevented pandaP461S to promote ventral fate strongly suggesting that PandaP461S signals through alk4/5/7 like a typical agonist.

*4. Lines 597-605: The discussion of the W-X-X-W motif that is part of the type I receptor binding motif in TGFß agonist and that is not conserved in Panda needs clarification. The authors state that this lack of conservation "suggests that Panda may also be affected in its ability to bind to the type I Receptor Alk4/5/7". Notably, the authors show that Panda does not bind to Type I BMP receptors but fail to test whether it binds to type I nodal receptors Alk4/5/7.*

The reviewer is right, this paragraph was confusing. The presence of a slightly divergent WXXW motif in Panda family members does not tell us much on the function of this motif or on the ability to bind to Alk4/5/7. Nevertheless, we think that the presence of a divergent WXXW motif in these factors argues in favor of the idea that these factors have a reduced ability to bind to and to activate Alk4/5/7. We have slightly modified the text for " This suggests that the Panda family of antagonists than includes Panda from urchins and Maverick from Drosophila may have a reduced ability to bind to the type I receptors."

*Minor recommendations:*

*1. Please double check all references for accuracy as they do not always match the text, which makes it difficult for non-urchin experts to gather needed information. For example lines 191-193: "it was first suggested that Panda might function as a BMP ligand for the BMP receptors Alk1/2 and Alk3/6 and therefore that Panda may antagonize Nodal signaling by promoting signaling from a pathway that is known to oppose Nodal signaling [15-19]." Only reference 17 relates to urchin Panda, others cite Smad based patterning in mouse or frog.*

We understand the comment of the reviewer and we have checked all the references as requested. We note however, that the two examples provided by the reviewer are accurately used. The set of references (15-19) was cited on purpose with ref 17 to illustrate that Panda antagonizes a BMP pathway that is known to oppose Nodal and the other references to illustrates studies showing that the BMP and Nodal pathways are antagonistic. All four are therefore useful.

*Lines 234-237: A recent study showed that Nodal can bind with high affinity to BMPRII, a type II receptor primarily used by BMPs, raising the possibility that Nodal may utilize both ACVRII and BMPRII to activate Nodal signaling [22]. It's not clear whether the authors are referring to urchin or vertebrate nodal receptors; Reference 22 cites studies of human Cerberus binding to nodal.*

Similarly, Ref 22, which cites a study on human Cerberus is the reference that should be cited to illustrate the fact that Nodal was found to bind BMPRII with high affinity.

*2. The sequence in Fig 4A cannot be deciphered. Since relevant sequence that is alluded to lies within the ß6-ß7 knuckle region, sequence upstream of this should be deleted and ß6-7 sequence enlarged enough to be readable.*

We have followed the recommendation of the reviewer. We deleted the sequences upstream of β6-β7 and enlarged the rest of the alignment to make it more readable.
